# Are E-Cigarette Flavors Associated with Exposure to Nicotine and Toxicants? Findings from Wave 2 of the Population Assessment of Tobacco and Health (PATH) Study

**DOI:** 10.3390/ijerph16245055

**Published:** 2019-12-11

**Authors:** Danielle M. Smith, Liane M. Schneller, Richard J. O’Connor, Maciej L. Goniewicz

**Affiliations:** 1 Department of Health Behavior, Roswell Park Comprehensive Cancer Center, Buffalo, NY 14263, USA; Liane.Schneller@RoswellPark.org (L.M.S.); richard.oconnor@roswellpark.org (R.J.O.); maciej.goniewicz@roswellpark.org (M.L.G.); 2 Clinical and Translational Science Institute, University of Rochester Medical Center, Rochester, NY 14642, USA

**Keywords:** Electronic Nicotine Delivery Systems (ENDS), flavorings, nicotine, biomarkers, Harmful or Potentially Harmful Constituents (HPHCs)

Increasing adoption of electronic cigarettes (e-cigarettes) has led to numerous concerns about health effects resulting from long-term use [1,2,3]. While many factors contribute to the popularity of these products [2], the availability of e-cigarettes in mint, fruit, sweet, and other appealing flavors is often cited as a reason for e-cigarette use, especially among youth and young adults [4,5,6,7,8]. Flavoring agents used in e-cigarettes are generally recognized as a safe (GRAS) for ingestion in most consumer products [9]. However, the inhalation toxicity and other potential health effects related to repeated inhalation of many of these flavoring agents remain largely unknown, and may range from acting as contributors to respiratory irritation, up through contributing to the development of systemic diseases [9]. Emerging evidence from in vitro and laboratory studies indicate that one of the most popular classes of flavorings present in e-cigarettes—fruit flavors [6,7,8,10,11]—has been linked to exposure to greater concentrations of known inhalation irritants [12], diminished bronchial epithelial cell metabolic activity and viability, and increased release of pro-inflammatory cytokines [13,14]. Importantly, laboratory findings have also implicated fruit-flavorings in potentially boosting the delivery of nicotine from e-cigarettes to the user relative to other e-cigarette flavorings [15,16], which may contribute to the addictive potential and abuse liability of these products. However, results found in laboratory studies commonly do not translate to observations from naturalistic settings, which merit the examination of this phenomenon using other data sources. Moreover, it is important to examine whether fruit-flavorings may also affect systemic concentrations of other toxicants present in e-cigarettes. Using nationally-representative data, we assessed whether the use of specific e-cigarette flavors was associated with select urinary biomarkers of exposure to nicotine and toxicants in regular users of e-cigarettes.

Using data from Wave 2 of the Population Assessment of Tobacco and Health (PATH) Study Biomarker Restricted Use Files [17,18], we analyzed levels of nicotine (biomarker: cotinine) and three select tobacco-related toxicants among exclusive e-cigarette users who reported using their product within the last 24 h (*n* = 211). Toxicant exposures examined in this analysis include acrylonitrile (biomarker: CYMA), benzene (biomarker: PMA), and acrolein (biomarker: CEMA), all of which present numerous health hazards (including respiratory irritation and carcinogenic potential), and have been linked to e-cigarette use [19]. Exclusive e-cigarette users reported their use of flavored e-cigarettes within the past 30 days, which were classified into use of (1) fruit-only, (2) tobacco-only, (3) single other flavor (including mint, clove, chocolate, and other reported flavors), and (4) fruit + use of additional flavors.

Due to the lognormal distribution of biomarker data, these outcomes were log-transformed to more readily approximate a normal distribution. Biomarkers with values under the limit of detection (LOD) were imputed by substituting the LOD/√2 [20]. To assess associations between use of flavored e-cigarettes and biomarker concentrations, creatinine-adjusted geometric means were calculated to account for potential differences in urine dilution [21], and differences in biomarker concentrations according to each flavor grouping were compared using simple linear regression models. Pairwise comparisons were conducted to assess between-flavor differences, and *p*-values were set at 0.05 and were adjusted for multiple comparisons using a Sidak correction. All analyses were weighted in accordance with procedures outlined in the PATH Biomarker Restricted Use File user guide [22], and were conducted using *svy* procedures in Stata v. 15.0.

The results of the analysis are displayed in Figure 1. Most exclusive e-cigarette users reported using only mint, clove, chocolate, and other reported flavors (31%), and fruit and additional flavors (31%), followed by tobacco-only (19%), and fruit-only (19%). Users of fruit-only flavored e-cigarettes exhibited significantly higher concentrations of the biomarker for acrylonitrile (CYMA) compared to users of a single other flavor (geometric mean ratio = 2.71, 95% CI: 1.30–5.62, adjusted *p*-value 0.048). Concentrations of biomarkers of exposure to nicotine (cotinine), benzene (PMA), and acrolein (CEMA) did not significantly differ across flavors.

Using population-based biomarker data, we did not confirm findings from laboratory studies suggesting that fruit-flavored e-cigarettes contribute to significantly elevated concentrations of nicotine among exclusive e-cigarette users. However, we did observe significantly greater concentrations of acrylonitrile among those who used a single e-cigarette flavor other than fruit or menthol. Differences in user behavior, devices, and e-liquids used likely to play a role in this discrepancy, and should be investigated in future studies on this topic. Considering these findings in light of these limitations and the context of existing evidence, future work should aim to further investigate the role that e-cigarette flavors may play in affecting user-health outcomes.

## Figures and Tables

**Figure 1:**
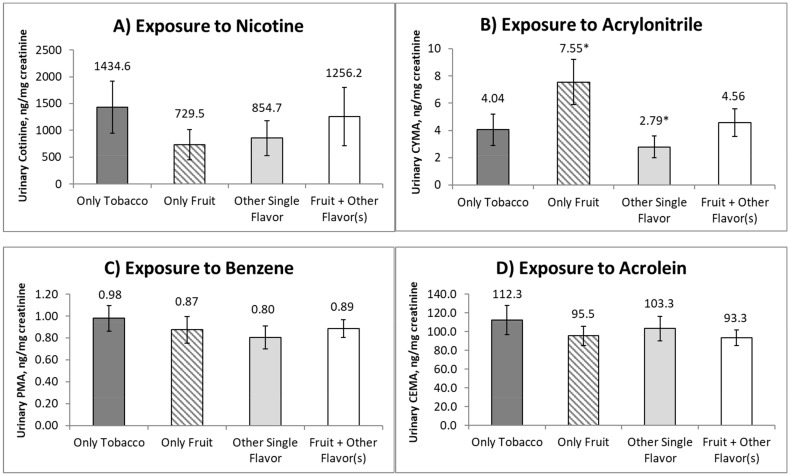
Urinary concentrations of biomarkers of exposure to (**A**) Nicotine, (**B**) Acrylonitrile, (**C**) Benzene, and (**D**) Acrolein, among exclusive users of flavored e-cigarettes, United States, 2015–2016 (*n* = 211). * indicates a statistically significant difference between flavors (Sidak-adjusted *p*-value < 0.05).
